# Cerebellar cavernous malformations with and without associated developmental venous anomalies

**DOI:** 10.1186/1471-2377-13-134

**Published:** 2013-10-03

**Authors:** Peifeng Zhang, Lingtong Liu, Yong Cao, Shuo Wang, Jizong Zhao

**Affiliations:** 1 Department of Neurosurgery, Shantou Center Hospital, 114 Waima Street, Shantou 515000, China; 2 Department of Neurosurgery, Beijing Tiantan Hospital, Capital Medical University, 6 Tiantan Xili, Chongwen District, Beijing 100050, China

**Keywords:** Cerebellum, Cavernous malformations, Developmental venous anomalies, Surgery, Prognosis

## Abstract

**Background:**

The clinical profiles of cerebellar cavernous malformations (CCMs) with and without associated developmental venous anomalies (DVAs) are not well known. The aims of this study were to analyze the clinical and radiological characteristics of CCMs and to assess the various therapeutic strategies.

**Methods:**

A consecutive series of 41 patients with identified CCMs were retrospectively reviewed. Of these, 11 patients (26.8%) were found to have associated DVAs. We compared the clinical profile of the two groups of patients (CCMs with and without DVAs). The CCMs with DVAs cases underwent radical resection of the CCMs, and the distal radicles of the DVAs that directly drain from the CCMs were coagulated and dissected at the length of the CCMs.

**Results:**

There were no statistically significant differences between the two groups with regard to age, sex, location and size of lesions, multiplicity, and surgical prognosis. The patients with CCMs with DVAs did not experience any brain swelling or hemorrhagic tendency intraoperatively. The postoperative course was uneventful for all of the 36 surgical patients with the exception of two of the patients with CCMs with associated DVAs, who suffered from serious cerebellar edema, and one of these two patients underwent an emergency suboccipital decompression craniotomy. With the exception of three patients who were lost to follow-up (mean, 22.3 months), all of the CCMs patients exhibited good long-term prognosis (modified Rankin scale values of 0–2) and no reoccurrence.

**Conclusions:**

It is not rare that associated DVAs occur in CCMs. The total removal of the CCM combined with the coagulation and dissection of the distal radicles of DVA at the length of the associated CCM may result in good long-term prognosis in patients.

## Background

The incidence rate of cavernous malformations (CMs) ranges from 0.4 to 0.9% of the general population. These incidences constitute 8 to 15% of all cerebrovascular malformations [1-5] and occur in the supratentorial compartment in 63 to 90% of cases [6,7]. Posterior fossa CMs represent 7.8 to 35.8% of all cases, and the brainstem is the most commonly involved site in this compartment (9-35% of all cases) [4,7]. Based on a large sample size, cerebellar CMs (CCMs) constitute 1.2 to 11.8% of all intracranial cases and 9.3 to 52.9% of all infratentorial cases [7,8]. The current data in the literature regarding CMs localized in the cerebellum are limited to case reports [7,9-12].

The coexistence of a CM and an associated developmental venous anomaly (DVA) is the most common mixed vascular malformation [13]. Patients with CMs associated with DVAs are more likely to have lesions in the posterior fossa [11]. The difference in the clinical profiles and surgical outcome between patients with CMs with and without associated DVAs in the cerebellum is unclear, which makes it difficult to determine the long-term prognosis of these patients.

There is controversy regarding the management of CMs associated with DVAs. Recent findings support the hypothesis that DVAs can be considered the initial pathophysiological factor that leads to the formation of CMs [14-18]. Additionally, some authors have proposed the coagulation and division of the transcerebral vein of the DVA during the removal of the CM as a means to prevent the recurrence of CMs [18]. However, due to the compromise of venous drainage of normal cerebellum, this strategy might increase the risk of serious postoperative cerebellar edema and might even cause cerebellar tonsillar herniation. The identification of a balance between the recurrence of the associated CM and herniation, which is a devastating consequence, currently remains unknown.

We retrospectively reviewed 41 patients suffering from cerebellar CMs with or without associated DVAs in our hospital to analyze the clinical and radiological characteristics of CCMs and to assess the various therapeutic strategies.

## Methods

### Patient population

Between February 2009 and April 2012, 456 consecutive patients (236 male and 220 female) harboring CMs involving the brain were hospitalized and treated at the Beijing Tiantan Hospital of Capital Medical University. Of these patients, 41 were diagnosed with CCMs. These CCM patients accounted for 8.99% of all of the brain CM patients. We excluded all of the supratentorial cases, all of the patients with CMs that were localized primarily in the brainstem and extended to the cerebellum, and all of the patients with CMs that were located at the cerebellopontine angle (CPA) and tentorium. The clinical data, including the patient’s age, sex, medical history, clinical presentation, radiological studies, and surgical notes, were reviewed and characterized. The study was approved by the Beijing Tiantan Hospital Review Committee. The patient information is summarized in Additional file 1: Table S1.

Each patient's clinical presentation was classified as incidental/asymptomatic or symptomatic hemorrhage (headache, vertigo, vomiting, gait disturbance, and ataxia). The diagnosis of symptomatic hemorrhage was made if there was a clinical history of acute neurological deterioration and either magnetic resonance imaging (MRI) or computed tomography (CT) evidence of associated acute hemorrhage.

The diagnosis of CM was established by MRI in 41 cases and by histopathological confirmation in 38 cases (36 cases of CCMs, one brain stem CM, and one temple CM). The diagnosis of CMs with or without associated DVAs was defined by MRI (41 cases) and/or by digital subtraction angiography (DSA) (9 cases) and subsequently confirmed by surgical findings (36 cases). MRI or DSA was used to define CMs and DVAs as per published criteria [19,20].

The size of the CM was measured using serial MRI or CT and/or intraoperative measurement.

### Surgical treatment

A CM was considered for surgical treatment if at least one of the necessary criteria described by Porter were fulfilled [4]. In total, 36 patients underwent surgery for the removal of CCMs. In addition, one of the two patients with multiple CMs underwent resection of a temple CM, and the other underwent resection of a brain stem CM. The other three patients were treated conservatively.

**Figure 1:**
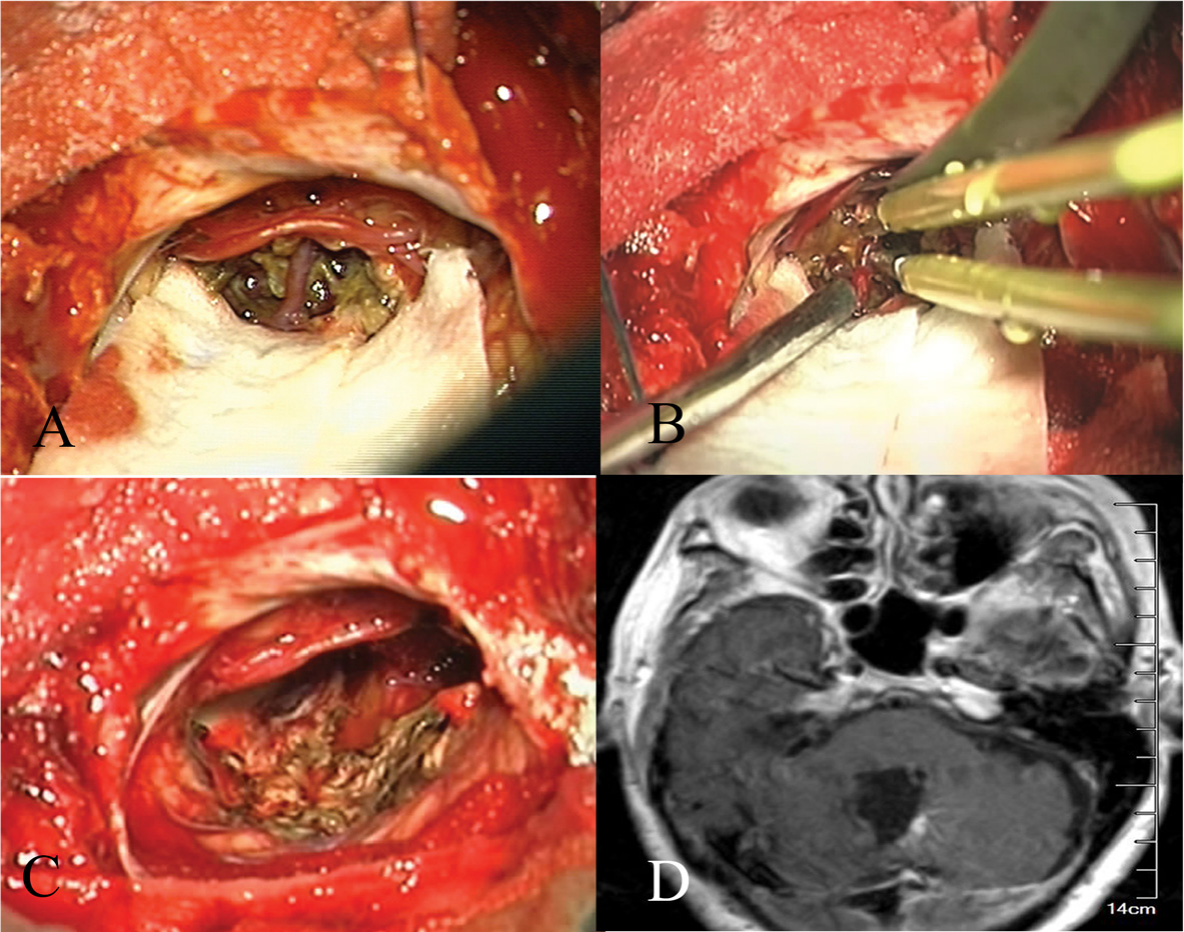
**CCMs with associated DVAs underwent total resection. A**. Intraoperative screenshot showing the CMs in the vermis and the radicle of the DVA before removal (same patient). **B**. Intraoperative screenshot showing that the radicle of the DVA was coagulated (same patient). **C**. Intraoperative screenshot showing that the CM was completely removed and that other radicles of the DVA draining from the surrounding normal brain were preserved (same patient). **D**. Postoperative MRI with contrast showing that the trunk and other radicles of the DVA draining from the surrounding normal brain were preserved after removal of the CM (same patient).

The pure CCMs were removed according to the standard microsurgical principle. In the cases of CCMs with associated DVAs, the CCMs underwent total resection, and the distal radicles of the DVAs, which directly drain from the CCMs, were coagulated and dissected at the length of the associated CM. The trunk of the DVAs that drained from the normal cerebellum was preserved. We did not attempt to excise the caput medusae, which lies within the normal cerebellum (Figure 1).

The morbidity and mortality were recorded. Any newly emerging neurological dysfunction or deterioration of a prior neurological deficit that appeared shortly after operation (within one week) was defined as a bad short-term result. No new neurological dysfunction or mitigation of an original neurological deficit after surgery was regarded as a good short-term result.

The most recent checkup was conducted in October 2012. The modified Rankin Scale (mRS) [21] was used to grade the long-term outcomes: mRS scores of 3–6 referred to bad long-term outcomes, and mRS scores of 0–2 referred to good long-term outcomes.

### Statistical analysis

The statistical comparisons were performed using Fisher's exact test for category variables or one-way ANOVA (analysis of variance) for continuous variables. All of the statistical analyses were performed using the SPSS 18.0 software (SPSS, Inc. Chicago, IL, USA). Differences with a P-value of less than 0.05 were considered statistically significant.

## Results

### Characteristics of the included population and comparison between CCMs with and without associated DVAs

The mean age of the 41 patients was 35.8 years (range 3 to 72 years). The ratio of males to females was 1.4:1. Of these 41 patients, 30 (73.2%) had pure CCMs without associated DVAs (CM group). The remaining 11 patients (26.8%) all had CCMs with associated DVAs (CM + DVA group). There was no statistically significant difference between the two groups regarding age, sex, location and size of lesions, multiplicity, initial clinical presentation, frequency of symptomatic hemorrhage, preoperative mRS, and surgical prognosis. Five (16.7%) of the 30 patients in the CM group and none of the 11 patients (0%) in the CM + DVA group had multiple CMs (Fisher's exact test, P = 0.30; Tables 1 and 2).

**Table 1:** Comparison between patients diagnosed with CCMs with and without associated DVAs (Fisher’s exact test)

	**CCMs**	**CCMs + DVA**	**P**
**Sex**			
Male	18(60%)	6(54.5%)	1.000
Female	12(40%)	5(45.5%)	
**Initial clinical presentation**			
Asymptomatic/incidental	0	0	1.000
Systematic bleeding	29(96.7%)	11(100%)	
Epilepsy	1(3.3%)	0	
**Location of CM**			
Cerebellar hemisphere	21(70%)	6(54.5%)	0.485
Vermis	8(26.7%)	4(36.4%)	
Cerebellar hemisphere + Vermis	1(3.3%)	1(9.1%)	
**Multiplicity of CMs**			
Multiple	5(16.7%)	0(0%)	0.300
Single	25(83.3%	11(100%)	
**Preoperative hemorrhage**			
Once	28(93.3%)	9(81.8%)	0.399
Twice	1(3.3%)	2(18.2%)	
Multiple	1(3.3%)	0(0%)	
**Pre-op mRS**			
0-2	27(90%)	11(100%)	0.551
3-6	3(10%)	0(0%)	
**#Post-op morbidity**			
Good	26(100%)	8(80.0%)	0.085
Bad	0(0%)	2(20.0%)	
▲**Follow-up mRS**			
0-2	23(100%)	10(100%)	NA*
3-6	0(0%)	0(0%)	

*Not applicable.

#Removal of cerebellar CM was performed in 36 patients.

▲Of the 36 patients, 33 participated in the follow-up, two were lost during the follow-up, and one died from an accident.

**Table 2:** Comparison between patients diagnosed with CCMs with and without associated DVAs (one-way ANOVA)

	**CCMs**	**CCMs + DVA**	**F**	**P**
**Mean age**	36.8	33.2	0.389	0.536
**Mean maximum lesion diameter (cm)**	2.053	2.018	0.012	0.913

### Patterns of relationship between CCMs and associated DVAs

**Figure 2:**
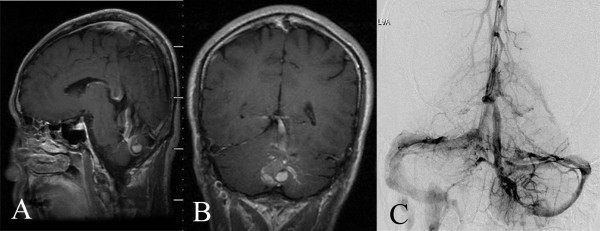
**Trunks of the DVAs passed upward and/or forward. A**, **B**. MRI with contrast showing that the trunk of the DVAs extended forward and upward to end in Galen’s vein. The distal radicles of the DVAs covered the anterior and superior aspect of the CMs. **C**. DSA showing that the trunk of the DVAs extended forward and upward to end in Galen’s vein.

**Figure 3:**
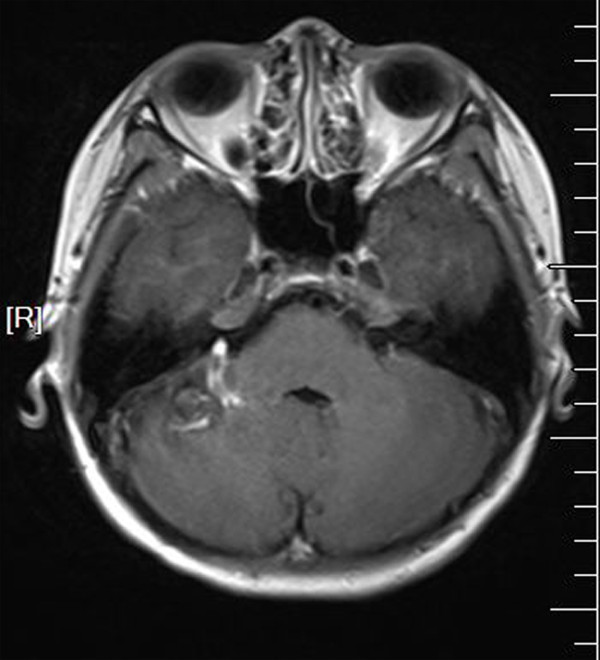
**MRI with contrast showing that the trunk extended upward and medial to the superior petrosal sinus.** The distal radicles of the DVAs covered the anterior, superior, and medial aspects of the CMs.

**Figure 4:**
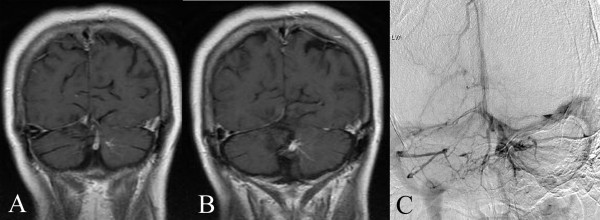
**Trunks of the DVAs extended upward. A**, **B**. MRI with contrast showing that the trunk extended upward and entered the proximal transverse sinus. The distal radicles of the DVAs covered the anterior and superior aspects of the CMs. **C**. DSA showing that the trunk extended upward and entered the proximal transverse sinus.

**Figure 5:**
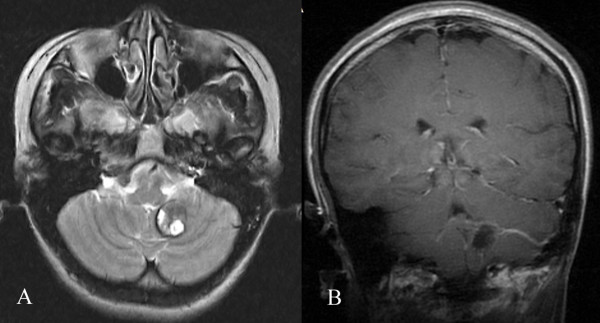
**Trunks of the DVAs extended forward and laterally. A**. Preoperative MRI T2 showing that the trunk extended forward and laterally to the sigmoid sinus. **B**. Postoperative MRI with contrast showing that the preserved trunk extended laterally to the sigmoid sinus.

According to the drainage endpoint of the DVAs, four patterns for the relationship between CCMs and associated DVAs were found in our study: the trunks of the DVAs passed upward and/or forward to end in the straight sinus or Galen’s veins (three cases; Figure 2), the trunks of the DVAs extended forward to the superior petrosal sinuses or inferior petrosal sinuses (four cases; Figure 3), the trunks of the DVAs extended upward and entered the proximal transverse sinus (two cases; Figure 4), and the trunks of the DVAs extended forward and laterally to the distal sigmoid sinus (two cases; Figure 5). We did not find the trunk of a DVA draining into the occipital sinus in any of the studied cases.

In the 11 cases analyzed in this study, the distal radicles of the DVAs usually covered the anterior aspect, the top of the CCMs. In no case did the distal radicles of the DVAs cover the bottom aspect of the CMs, and the trunk of the DVAs never extended downward.

### Surgical outcome

**Figure 6:**
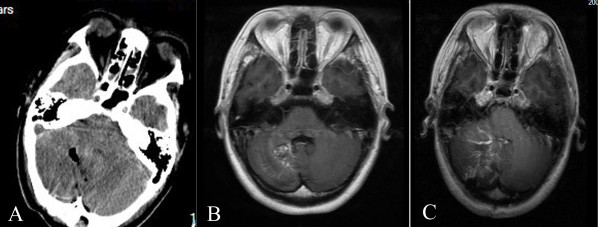
**Radiographic study in patient 11. A**. CT showing serious cerebellar edema one day after surgery. **B**. Preoperative MRI with contrast showing the CMs and associated DVAs. **C**. Postoperative MRI with contrast showing cerebellar edema and that the other radicles of the DVA remained after removal of the CM.

**Figure 7:**
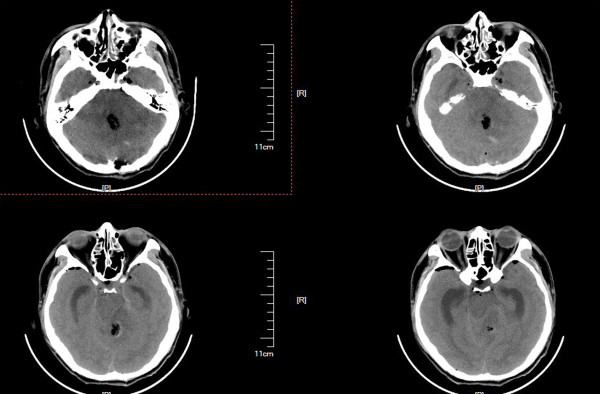
CT showing serious cerebellar edema and supratentorial hydrocephalus.

All of the 36 patients with CCMs were treated surgically using standard microsurgical approaches. The resection of CCMs was radical and was documented and confirmed by intraoperative ultrasound and postoperative MRI. The 10 associated DVAs were confirmed intraoperatively, and the distal radicles of these DVAs were coagulated and dissected at the length of the associated CMs. No brain swelling or hemorrhagic tendency was encountered intraoperatively. The postoperative course was uneventful for all of the CCM patients except two CCM + DVA patients (patient 11 and patient 35). One day after surgery, patient 11 was conscious but complaining of a progressively worsening headache and vomiting. The CT scans showed a serious cerebellar edema (Figure 6). Conservative treatment, including mannitol dehydration and methylprednisolone to decrease the intracranial pressure (ICP), was administered to the patient. One week later, the patient returned to normal status. Patient 35 experienced dizziness 12 hours after surgery, and the CT scans showed serious cerebellar edema and a supratentorial hydrocephalus (Figure 7). An emergency frontal horn external drainage was performed, and subsequent conservative treatment for lowering the ICP (mannitol dehydration and methylprednisolone) was administered. These treatments resulted in the patient regaining consciousness. However, three days later, the patient fell into a coma. A CT scan showed that the cerebellar edema had become more serious; thus, an emergency suboccipital decompression craniotomy and clearance of the surrounding cerebellar necrotic tissue was performed. During the procedure, the trunk vein of the DVA was found to be already occluded. Ten days later, the patient regained consciousness. In total, none of the 36 surgical patients died. The short-term results exhibited no statistically significant difference between the two groups (Table 1).

### Follow-up

The clinical follow-up period ranged from 6 months to 42 months (38 patients; mean 22.3 months), and the radiographic follow-up (MRI and/or CT) period ranged from 6 months to 41 months (38 patients; mean 17.7 months). Of the 36 surgical patients, 33 attended the follow-up, two were lost, and one died from an accident. All of the patients in both the CM and the CM + DVA groups had good long-term prognosis, and there was no statistically significant difference between the two groups (Table 2). No CCM reoccurrence or rehemorrhage was observed in the 33 patients. Of the five conservatively treated CCM patients, one patient (patient 1) suffered a cerebellar lesion and minor enlargement, and we have continued to observe the patient’s progress.

## Discussion

Intracranial vascular malformations were originally classified by McCormick [22] into five types: telangiectasias, varix, cavernous malformation, arteriovenous malformation, and venous malformation. The definition of each of these types of malformations depends on distinct pathological criteria. However, mixed or transitional vascular malformations with the pathological features of more than one type of malformation within the same lesion have been recently described. The detection of a wide spectrum of intermediate forms of McCormick’s original categories suggests that these lesions might be a continuum of progression from a single pathological process [12]. The coexistence of a CM and a DVA is the most common presentation of a mixed vascular malformation. Much discrepancy exists in the literature concerning the prevalence of coexisting CMs and DVAs [13]. Based on findings from MR imaging studies, Abdulrauf [11] found that 13 (24%) of 55 patients with supratentorial and infratentorial CMs also had associated DVAs. A similar percentage was reported by Wurm [18], who found this type of association in 15 (25.9%) of 58 patients with cerebral, cerebellar, and brain stem CMs based on MR imaging and intraoperative findings. In 86 surgically treated patients with brainstem CMs, Porter [4] found a DVA that was intimately associated with each resected CM. Oliveira [7] reported cerebellar CMs in 10 cases, but none of these were associated with a DVA. In our study, 11 cerebellar CMs (26.8%) with associated DVAs were identified in 41 consecutive cerebellar CM patients.

Abdulrauf [11] compared the clinical profile of patients harboring CMs with and without associated DVAs. Compared with patients with CMs alone, patients with CMs associated with DVAs are more likely to be female, have associated symptomatic hemorrhage, have lesions in the posterior fossa (statistically significant), and suffer from repeated symptomatic hemorrhage and are less likely to present with seizures or to have familial histories. The authors of several reports have suggested that the CMs associated with a DVA have a more aggressive clinical course than CMs alone [11,14,15,23]. In our study, the CCMs associated with DVAs did not present a more aggressive nature. There were no statistically significant differences between the patients with CCMs with and without DVAs regarding age, sex, location and size of the lesions, and initial clinical presentation. Five of the 30 patients (16.7%) in the CM group and none of the 11 patients (0%) in the CM + DVA group had multiple CMs (P = 0.30, not statistically significant). Familial cerebral CMs caused by loss-of-function mutations are often characterized by multiple lesions [24]. If substantiated in a larger case series, this difference of multiplicity may suggest different pathogenesis mechanisms (i.e., genetic factors, structural venous outflow factors, and hemodynamic variables) of the host’s predisposition to CCMs with and without associated DVAs.

The association of CMs and DVAs within the same lesion has generated hypotheses about the causation–evolution relationship among different types of malformations. It has been postulated that the abnormal hemodynamics of DVAs might induce the formation of CMs [12]. It has also been suggested that chronically increased intraluminal pressure and the resulting reduced tissue perfusion leading to tissue hypoxia may stimulate a local increase in angiogenic factors, which would induce the formation of vascular malformations [17]. If there are mixed vascular malformations in a region, DVAs can be considered the primary congenital lesion, and venous hypertension can be regarded as the initial pathophysiological factor leading to the formation of CMs [14,17,18,25].

At the same time, DVAs are also responsible for the venous drainage of otherwise normal brain tissue. The removal of the whole DVA to prevent the reoccurrence of the associated CM could result in venous engorgement and cerebral edema, which might have devastating consequences. As a result, the following treatment protocol is recommended for patients with CMs associated with DVAs to avoid the risk of venous infarction: microsurgical resection of the CM alone and an “untouching” strategy for the DVA [4,10,16].

These two vascular malformations are usually connected by small medullary veins. The concurrent CM is located in the territory of the DVA, and the DVA’s distal radicles are part of the CM. The untouching of the DVA is difficult and even unrealistic unless residual CM is left in the region, which might result in CM reoccurrence and rehemorrhage.

The authors of some surgical reports have challenged this prevalent thinking and have described a significantly improved operative treatment of DVAs [12]. In a recent provocative report, Wurm [18] proposed the coagulation and division of the transcerebral vein of the DVAs to prevent CM recurrence. In their series of 15 patients with DVAs and CMs, these researchers removed the CM and divided the transcerebral vein of the DVA in nine of the patients: in six during the first operation and in the remaining three patients after recurrence of the associated CM with symptomatic hemorrhage. These authors observed no brain swelling intraoperatively, and the postoperative course was uneventful in all of these nine patients. Their experience supports the theory that the abnormal draining vein might be the actual pathological lesion that causes blood flow disturbances with recurrent and newly developing malformations. Coagulation of the large transcerebral draining vein did not lead to any ischemic or hemorrhagic infarction or any other complication.

The four patterns of relationship between CCMs and associated DVAs found in our study were summarized. The distal branches of the DVAs always covered the anterior top aspects of the CM, and the trunk of the DVA is more likely to extend upward and forward. In no cases did the distal radicles of the DVA cover the bottom aspect of the CM, and the trunk of the DVA never extended downward. In most surgical cases, the cerebellum is exposed from the back and lower head; thus, the relationship patterns of the CCMs and associated DVAs provide the surgeon great convenience to reach the CM and to coagulate and dissect the connected radicles without the barrier of the trunk. This surgical strategy might markedly aid the safe and radical removal of CMs and prevent the reoccurrence of CMs, and these patients may also have good long-term prognosis. It has to be emphasized that the surgeon must be alert when completing the resection of the CM because the trunk of the DVA is usually hidden behind the CM. In two of the patients analyzed in our study, the trunks of the DVAs might have been injured during the dissection of the anterior aspect and top aspect of the CMs, which would cause serious and dangerous cerebellar edema.

### Limitations

In our series, the results preliminarily showed similarity in the clinical characteristics and surgical prognosis between CCMs associated or not associated with DVAs. A more definitive conclusion would require a larger sample size, randomized clinical trial, and a longer prospective follow-up. In addition, it must be noted that the postoperative incidence of early serious cerebellar edema in the CCMs with associated DVAs in our series was much higher than that obtained with the pure CCM cases (20% vs. 0%), but this different was not statistically significant. Further research is necessary to determine whether the operation of CCMs with associated DVAs is associated with higher early morbidity.

## Conclusions

It is not rare that associated DVAs are found in CCM cases. There were no statistically significant differences between the patients with pure CCMs and the patients with CCMs with associated DVAs with respect to age, sex, location and size of lesions, multiplicity, initial clinical presentation, frequency of symptomatic hemorrhage, preoperative mRS, and surgical prognosis. The total removal of the CCM combined with the coagulation and dissection of the distal radicles at the length of the associated CM and the preservation of the trunk of the developmental venous anomalies can result in good long-term prognosis in patients.

## Consent

Written informed consent was obtained from the patient for the publication of this report and any accompanying images.

## Competing interests

The authors declare that they have no competing interests.

## Authors’ contributions

Dr. PZ is responsible for the collection of the clinical data, the analysis of the radiological study, and drafting the article. Dr. LL is responsible for the collection of the clinical data, the statistical analysis of the data, and drafting the article. Dr. YC is responsible for the design of the study, the surgical treatment of the patients, the interpretation of the clinical data, and critically revising the draft for important intellectual content. Dr. SW is responsible for the design of the study, the surgical treatment of the patients, the supervision of the research, critically revising the draft, and the final approval of the version to be published. Prof. JZ is responsible for the surgical treatment of the patients, the supervision of the research, critically revising the draft, and the final approval of the version to be published. All authors read and approved the final manuscript.

## Pre-publication history

The pre-publication history for this paper can be accessed here:

http://www.biomedcentral.com/1471-2377/13/134/prepub

## Supplementary Material

Additional file 1: Table S1 Summary of patients. Click here for file
