# Supplementary material for: Cerebellar cavernous malformations with and without associated developmental venous anomalies
Source: BMC Neurol. 2013 Oct 3;13:134. doi: 10.1186/1471-2377-13-134 (PMC3850546; doi:10.1186/1471-2377-13-134)
Supplement: Additional file 1: Table S1 — Summary of patients. [file 1471-2377-13-134-S1.doc]

Supplement table 1 Summary of patients

| NO | Initial clinical presentations | Location of CM | Maximum lesion diameter(CM) | Multiplicity of CMs | Frequency of symptomatic hemorrhage | Radiology | Drainage vein | Pre-op mRS | Surgical approach | Intraoperative finding confirmation | Duration of follow-up  (mo) | Post-op mRS | Follow-up mRS | Recurrence |
| --- | --- | --- | --- | --- | --- | --- | --- | --- | --- | --- | --- | --- | --- | --- |
| 1 | Headache  vertigo  vomiting | R Tempo lobe; L cerebellar hemisphere | 1.5 | Yes | 1 | CT, MRI | No | 1 | Frontal-temporal approach | NA | 42 | NA | NA | Yes* |
| 2 | Headache  Vertigo  Gait disturbance | Supetentorial r multiple; Brain stem; R cerebellar hemisphere , | 2.4 | YES | 1 | CT, MRI | No | 2 | Rt temporo-occipital approach for brain stem CM | NA | 42 | NA | NA | No |
| 3 | Vertigo | R cerebellar hemisphere | 4 | NO | 1 | CT | No | 1 | far lateral approach | No DVA | 42 | NA | Death of accident | NA |
| 4 | Headache  Vertigo  Vomiting | Vermis | 1.9 | No | 1 | CT | No | 1 | P.M.A | No DVA | 42 | NA | lost | NA |
| 5 | Vertigo  Vomiting | R cerebellar hemisphere | 0.7 | No | 1 | CT,MRI | No | 2 | P.M.A | No DVA | 41 | NA | 0 | No |
| 6 | Headache  Vertigo  Vomiting  Ataxia | L cerebellar hemisphere +Vermis | 5 | No | 1 | MRI | No | 1 | P.M.A | No DVA | 40 | 0 | 0 | No |
| 7 | Vertigo  Gait disturbance Vomiting | R cerebellar hemisphere | 1.7 | No | 1 | CT, MRI | No | 1 | lateral median approach | No DVA | 39 | 1 | lost | NA |
| 8 | Headache  Vertigo  Gait disturbance Vomiting | R cerebellar hemisphere | 1.2 | No | 2 | CT,MRI | No | 2 | P.M.A | No DVA | 39 | 0 | 0 | No |
| *9* | *Vetigo* | *L cerebellar hemisphere* | *3* | *No* | *1* | *MRI,DSA* | *L sigmoid sinus* | *1* | *PMA* | *DVA* | *38* | *1* | *0* | *No* |
| 10 | Gait disturbance vomiting | Vermis | 2 | No | 1 | MRI | No | 2 | PMA | No DVA | 38 | 0 | 0 | No |
| *11* | *Vertigo* | *R cerebellar hemisphere* | *1.4* | *No* | *1* | *MRI* | *Rt inferior petro sinus* | *2* | *P.M.A* | *DVA* | *35* | *3* | *2* | *No* |
| 12 | Vertigo  Gait disturbance | Vermis | 2.2 | No | 3 | CT,MRI,DSA | No | 2 | P.M.A | No DVA | 32 | 2 | 1 | No |
| 13 | Vertigo  Gait disturbance | Vermis | 3 | No | 1 | MRI | No | 2 | P.M.A | No DVA | 30 | 2 | 1 | No |
| *14* | *Headache* | *R cerebellar hemisphere* | *1.5* | *No* | *2* | *CT, MRI* | *Rt sup petro sinus* | *1* | *Retrosigmoid approach* | *DVA* | *27* | *1* | *1* | *No* |
| 15 | Headache  Vertigo  Vomiting | Vermis | 2.9 | No | 1 | MRI | No | 1 | PMA | No DVA | 27 | 1 | 1 | No |
| 16 | Headache  Vertigo  Gait disturbance | R cerebellar hemisphere | 2,9 | No | 1 | CT,MRI | No | 3 | PMA | No DVA | 25 | 2 | 2 | No |
| 17 | Vertigo  Vomiting | R cerebellar hemisphere | 2 | no | 1 | CT,DSA | No | 1 | Retrosigmoid approach | No DVA | 24 | 1 | 1 | No |
| 18 | Headache | Vermis | 1 | No | 1 | CT, MRI | No | 1 | P.M.A | No DVA | 22 | 0 | 0 | No |
| 19 | Vertigo  Gait disturbance | L cerebellar hemisphere | 1.6 | YES | 1 | MRI | No | 1 | P.M.A | No DVA | 19 | 1 | 1 | No |
| 20 | Vertigo  Gait disturbance Vomiting | R cerebellar hemisphere | 1.3 | No | 1 | MRI | No | 2 | P.M.A | No DVA | 18 | 2 | 1 | No |
| 21 | Vertigo | L cerebellar hemisphere | 2.5 | No | 1 | CT, MRI | No | 1 | Retrosigmoid approach | No DVA | 18 | 1 | 0 | No |
| 22 | Epilepsy | R Frontal lobe; multiple cerebellar hemisphere | 1.4 | Yes | 1 | CT, MRI | No | 1 | No surgery | NA | 18 | NA | NA | No |
| 23 | Headache  Vertigo  Gait disturbance | Vermis | 2.8 | No | 1 | CT, MRI | No | 2 | occipital transtentorial approach | No DVA | 18 | 2 | 2 | No |
| 24 | Headache  Vertigo | R cerebellar hemisphere | 0.6 | NO | 1 | MRI,DSA | No | 2 | No surgery | NA | 18 | NA | NA | No |
| 25 | Headache  Vertigo  Vomiting | L cerebellar hemisphere | 1.4 | No | 1 | CT, MRI | No | 1 | P.M.A | No DVA | 17 | 0 | 0 | No |
| 26 | Vertigo,  Vomiting | L cerebellar hemisphere | 1.7 | No | 1 | MRI | No | 1 | Retrosigmoid approach | No DVA | 16 | 1 | 2 | No |
| 27 | *Vertigo* | *Vermis* | *3* | *No* | *1* | *MRI* | *R sup petro sinus* | *2* | *PMA* | *DVA* | *15* | *1* | *2* | *No* |
| 28 | Headache  Vertigo  Vomiting | Supratentorial; cerebellar hemisphere | 2.3 | YES | 1 | MRI | No | 2 | P.M.A | No DVA | 13 | 2 | 2 | No |
| 29 | Headache  Vertigo  Gait disturbance | L cerebellar hemisphere | 3 | No | 1 | CT, MRI | No | 2 | P.M.A | No DVA | 13 | 0 | 0 | No |
| *30* | *Headache*  *Vertigo*  *Vomiting* | *Vermis* | *2.6* | *No* | *2* | *CT, MRI,DSA* | *Lt Transverse sinus* | *1* | *P.M.A* | *DVA* | *13* | *2* | *2* | *No* |
| *31* | *Vertigo*  *Gait disturbance* | *L cerebellar hemisphere* | *2.2* | *No* | *1* | *MRI* | *Lt sigmoid sinus* | *2* | *P.M.A* | *DVA* | *12* | *2* | *0* | *No* |
| 32 | Vertigo  Gait disturbance | L cerebellar hemisphere | 1.4 | No | 1 | MRI | No | 2 | P.M.A | No DVA | 11 | 2 | 2 | No |
| 33 | Headache,  Gait disturbance  Vomiting  Clumsy in hands | R cerebellar hemisphere | 1.7 | No | 1 | MRI | No | 2 | P.M.A | No DVA | 11 | 0 | 0 | No |
| *34* | *Headache* | *L cerebellar hemisphere* | *1.5* | *No* | *1* | *CT,MRI,*  *DSA* | *straight sinus* | *1* | *P.M.A* | *DVA* | *11* | *1* | *1* | *No* |
| *35* | *Vertigo,* | *L cerebellar hemisphere* | *1.5* | *No* | *1* | *CT, MRI* | *straight sinus* | *1* | *PMA* | *DVA* | *9* | *5* | *2* | *No* |
| *36* | *Vertigo*  *Gait disturbance Vomiting*  *Clumsy in left hand* | *Vermis* | *1.5* | *No* | *1* | *MRI, DSA* | *Lt inferior petro sinus* | *2* | *No surgery* | *NA* | *7* | *NA* | *NA* | *No* |
| 37 | Vertigo  Gait disturbance  Vomiting  Ataxia | R cerebellar hemisphere | 1.4 | No | 1 | MRI | No | 3 | PMA | No DVA | 7 | 1 | 1 | No |
| *38* | *Headache*  *Vertigo*  *Gait disturbance* | *L cerebellar hemisphere + Vermis* | *1* | *No* | *1* | *MRI, DSA* | *Galen’s vein* | *2* | *P.M.A* | *DVA* | *7* | *0* | *0* | *No* |
| *39* | *Vertigo* | *Vermis* | *3* | *No* | *1* | *MRI, DSA* | *Lt Transverse Vein* | *2* | *P.M.A* | *DVA* | *6* | *2* | *2* | *No* |
| 40 | Gait disturbance Vomiting | Vermis | 1.6 | No | 1 | MRI | No | 1 | P.M.A | No DVA | 6 | 1 | 2 | No |
| 41 | Vertigo  Gait disturbance  Vomiting  Ataxia | R cerebellar hemisphere | 2.5 | No | 1 | MRI | No | 3 | Retrosigmoid approach | No DVA | 6 | 1 | 2 | No |

PMA: Para Midline Approach; Sup petro sinus: superior petrosal sinus; *:cerebellar lesion enlargement; NA: not applicable. CCM+DVA group is in italic type..
